# Key carboxylate residues for iron transit through the prokaryotic ferritin *Syn*Ftn

**DOI:** 10.1099/mic.0.001105

**Published:** 2021-11-26

**Authors:** Justin M. Bradley, Joshua Fair, Andrew M. Hemmings, Nick E. Le Brun

**Affiliations:** ^1^​ Centre for Molecular and Structural Biochemistry, School of Chemistry, University of East Anglia, Norwich, NR4 7TJ, UK; ^2^​ Centre for Molecular and Structural Biochemistry, School of Biological Sciences, University of East Anglia, Norwich, NR4 7TJ, UK

**Keywords:** ferritin, iron, transport, oxidation, storage

## Abstract

Ferritins are proteins forming 24meric rhombic dodecahedral cages that play a key role in iron storage and detoxification in all cell types. Their function requires the transport of Fe^2+^ from the exterior of the protein to buried di-iron catalytic sites, known as ferroxidase centres, where Fe^2+^ is oxidized to form Fe^3+^-oxo precursors of the ferritin mineral core. The route of iron transit through animal ferritins is well understood: the Fe^2+^ substrate enters the protein via channels at the threefold axes and conserved carboxylates on the inner surface of the protein cage have been shown to contribute to transient binding sites that guide Fe^2+^ to the ferroxidase centres. The routes of iron transit through prokaryotic ferritins are less well studied but for some, at least, there is evidence that channels at the twofold axes are the major route for Fe^2+^ uptake. *Syn*Ftn, isolated from the cyanobacterium *Synechococcus* CC9311, is an atypical prokaryotic ferritin that was recently shown to take up Fe^2+^ via its threefold channels. However, the transfer site carboxylate residues conserved in animal ferritins are absent, meaning that the route taken from the site of iron entry into *Syn*Ftn to the catalytic centre is yet to be defined. Here, we report the use of a combination of site-directed mutagenesis, absorbance-monitored activity assays and protein crystallography to probe the effect of substitution of two residues potentially involved in this pathway. Both Glu141 and Asp65 play a role in guiding the Fe^2+^ substrate to the ferroxidase centre. In the absence of Asp65, routes for Fe^2+^ to, and Fe^3+^ exit from, the ferroxidase centre are affected resulting in inefficient formation of the mineral core. These observations further define the iron transit route in what may be the first characterized example of a new class of ferritins peculiar to cyanobacteria.

## Introduction

Iron is an essential micronutrient and, despite being the fourth most abundant element in the Earth’s crust, its availability is often a growth-limiting factor in microbes [1]. Many proteins involved in important biological processes such as respiration, nucleic acid synthesis, nitrogen fixation, sulphur cycling and oxygen activation require iron or iron containing species as cofactors [2–6]. Correct metallation of these proteins requires a labile pool of iron be maintained within the cytoplasm [7], the concentration of which is estimated to be of the order of 10 µm in most instances [8–10]. Much of the biological importance of iron is based on its ability to cycle between the Fe^2+^ and Fe^3+^ oxidation states, but this same redox activity presents a significant challenge in aerobic environments. In all but acidic conditions iron is readily oxidized by O_2_ to form insoluble ferric oxy-hydroxide minerals, which severely limits its bioavailability [11]. Furthermore the cycling of iron between the Fe^2+^ and Fe^3+^ states catalyses the formation of extremely toxic hydroxyl radicals from peroxide and superoxide produced as byproducts of aerobic respiration [12]. Therefore the redox chemistry of iron, essential for its biological role, imposes on all aerobically respiring organisms the dual challenges of acquiring iron at concentrations many orders of magnitude greater than those in the immediate environment, and ensuring the cellular concentration of free iron is tightly regulated to mitigate the potential for oxidative damage. Ferritins play an important role in this across all kingdoms of life by providing a repository of iron in a chemically inert form that can be accessed to fulfil metabolic need under conditions of iron limitation [13, 14].

Ferritins are hollow rhombic dodecahedral assemblies composed of 24 protomers, each of which adopts a 4 α-helical fold with a shorter fifth helix running perpendicular to the others [15]. Ferritins isolated from animals contain a tissue-dependent mixture of H-chains that harbour a ferroxidase centre (FOC), the catalytic site for oxidation of Fe^2+^, and l-chains that do not. The H- and l-chains are isostructural and co-assemble into 24meric heteropolymers [16]. In contrast, the ferritins of plants and microbes are composed solely of H-chain-like subunits [17]. Access to the interior of the cage is provided by channels penetrating the protein coat at the 4-, 3- and, in some instances, twofold symmetry axes of the dodecahedral assembly. In all H-chain-like ferritins the FOC is buried at the centre of the 4 α-helical bundle, a distance of at least 13 Å from the inner surface exit of any of the channels mentioned above. Ferritin function therefore requires a means to rapidly transfer incoming Fe^2+^ substrate from the channel exit to the FOC. Despite variability in the route via which Fe^2+^ substrate traverses the protein coat, it appears to be a common feature of all ferritins that its transfer from inner surface exit to FOC is facilitated by networks of flexible carboxylate residues located on the inner surface [18–22].

**Fig. 1.**
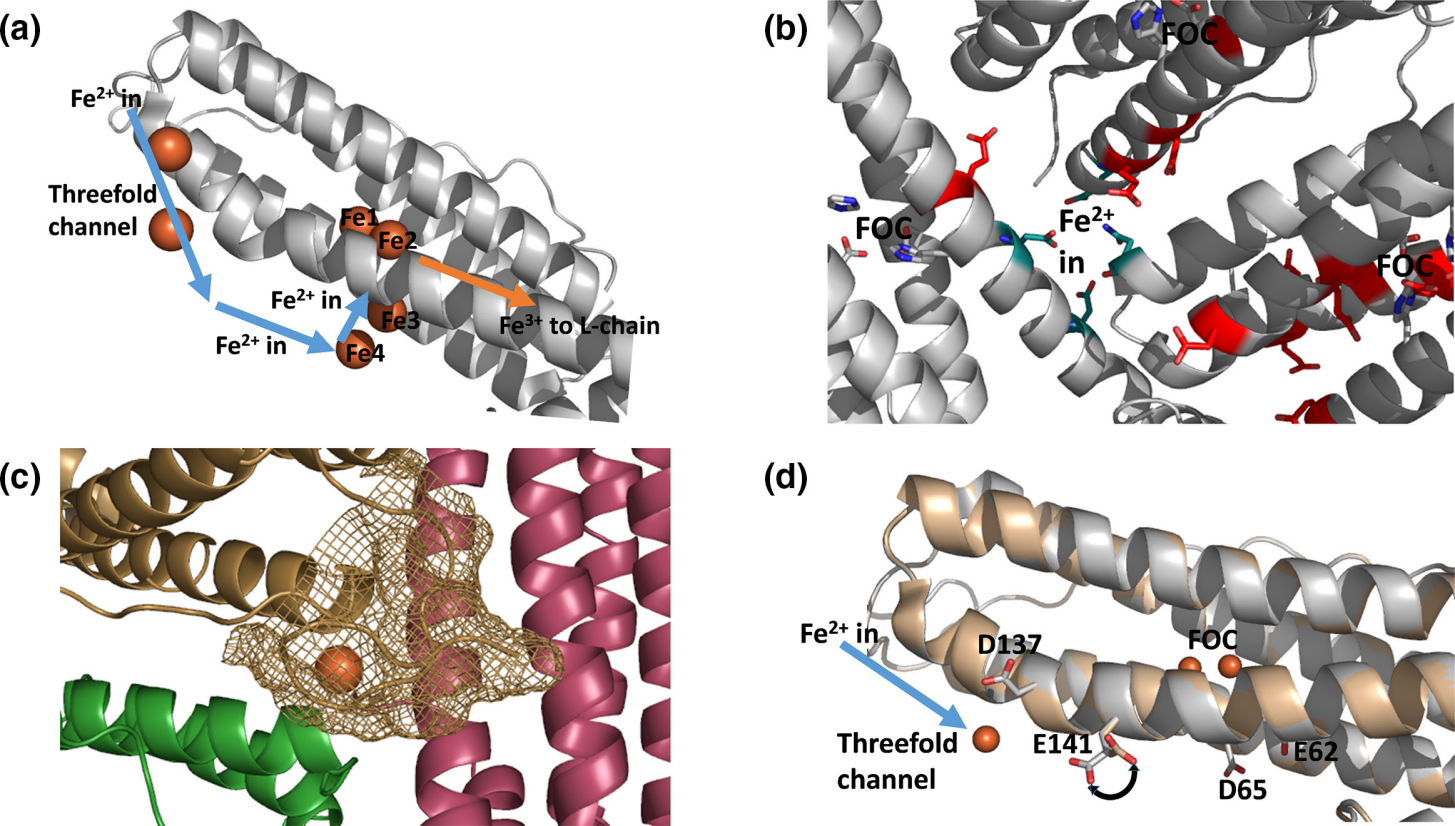
Iron entry to ferritins. (a) Iron enters the animal ferritins via the threefold channel before passing sequentially through sites 4 and 3 en route to the FOC. Oxidized product is thought to exit the FOC in the direction of the fourfold channel (indicated by the orange arrow) and is passed to the l-chain where mineralization is initiated at nucleation sites. (b) The B-channel of Bfr (highlighted in teal) viewed from the interior of the protein cage. The channel lies at the twofold symmetry axis at the interface between three protein monomers. Acidic residues that lie between the B-channel and each of the three associated FOCs are highlighted in red. (c) The B-channel of SynFtn overlaid with the position of Fe^2+^ located in the channel of PmFtn isolated from the pennate diatom Pseudonitzia multiseries [27]. Each monomeric unit forming the B channel is coloured differently. The brown mesh illustrates the surface associated with the N-terminal extension of the SynFtn monomer coloured brown which occludes the aperture of the B-channel on the outer surface of the protein. (d) Conformational flexibility in SynFtn residue Glu141. In the structure of the metal-free protein (shown in wheat) Glu141 is oriented toward the FOC. Soaking crystals in a 5 mm Fe^2+^ solution for 20 min (shown in white) results in occupancy of site 3 in the threefold channel and a change in conformation of Glu141 such that it is oriented toward this. The sidechains of other carboxylates thought to be important for iron transit in SynFtn are shown as sticks.

The route of iron transit through animal ferritins has been determined in greater detail than for other classes and appears to be common to all examples. The channel at the threefold pore is lined by a conserved CDFXE motif (where X=I or L) and constitutes the major entry route for Fe^2+^ substrate [23]. Two transient binding sites located on the inner surface of the protein then shuttle the Fe^2+^ to the FOC [24, 25] (Fig. 1a). There appears to be some variability in the amino acid residues that make up these transient binding sites, but all animal ferritins contain two conserved glutamate residues, E61 and E140 (numbered according to the position in the peptide sequence of the human H-chain subunit), that are involved in transport of Fe^2+^ to FOC sites [21]. Following oxidation, the Fe^3+^ product exits the FOC, enabling multiple turnovers and is transported to a site on the inner surface of the l-chain where mineral core nucleation occurs [26].

Whilst iron transit through the ferritins of prokaryotes has been less extensively studied, the B-channels, located at the twofold symmetry axes, have been shown to be the major route for iron entry where studied [19, 27]. Acidic residues line the inner surface in a route from the B-channel to the FOC (Fig. 1b) but their importance for function remains to be demonstrated [19]. Two distinct classes of ferritin have been identified in prokaryotes, the haem-binding Bfrs and the haem-free Ftns, and these appear to differ in their mechanism of mineral core formation. In the Ftns, three conserved Glu residues form a third iron binding site, known as site C, and in some examples this serves as the nucleation site for core formation [28]. The FOC architecture of Bfrs is unique amongst ferritins, in that the coordination of iron is similar to that in diiron monooxygenases such as ribonucleotide reductase [29] and they exhibit variability in the mechanism of core formation. In some, the FOC functions as a stable cofactor, continually cycling between di-Fe^2+^ and di-Fe^3+^ forms, which drives oxidation of Fe^2+^ in the protein cavity [30, 31]. In other examples, conformational flexibility of one of the FOC amino acid side chain ligands has led to the proposal of a ‘trap door’ mechanism in which oxidized product is released from the FOC directly into the interior of the protein cage [32].

*Syn*Ftn is a recently characterized ferritin isolated from the coastal dwelling cyanobacterium *Synechococcus* CC9311, which is unusual is several respects. It lacks the carboxylate residues that form site C in other Ftn proteins, and has been demonstrated to oxidize iron at the FOC via a mechanism distinct from that reported for any other ferritin to date [33]. The peptide sequence of *Syn*Ftn contains an N-terminal extension relative to other Ftns and this occludes the outer entrance of the B-channel (Fig. 1c), suggesting a novel route of iron uptake in this protein. Indeed it was recently demonstrated that Fe^2+^ enters *Syn*Ftn via the threefold channels [18] despite the absence of one of the carboxylates conserved in and essential to the equivalent channel in the animal ferritins. Furthermore, the conserved carboxylates of the transient binding sites of animal ferritins are absent from the *Syn*Ftn peptide sequence. Since *Syn*Ftn is a homopolymer of H-chain like units there is no possibility for core nucleation to be initiated at the inner surface of l-chains as occurs in animal ferritins. Therefore, it appears that, in addition to the unusual iron-oxygen chemistry of its FOC, *Syn*Ftn utilises a route of iron transit from exterior to mineral core that is distinct from any ferritin reported to date.

Crystal structures of *Syn*Ftn have been reported in the iron-free state and following either 2 or 20 min of soaking in a 5 mm Fe^2+^ solution. These revealed conformational flexibility of the side chain of residue Glu141, which is located close to the inner surface exit of the threefold channel. In the metal-free and 2 min iron soak structures, Glu141 is oriented towards the FOC. However, in the 20 min iron soak structure, an iron-binding site in the threefold channel, site 3, is occupied and Glu141 is oriented toward this (Fig. 1d), with the distance from the ε2 oxygen of Glu141 to site 3 reduced from 8.9 to 5.9 Å. This change in conformation in response to the iron status of the protein is precisely the behaviour expected for a residue involved in transfer of substrate to the FOC. To probe the possibility that Glu141 of *Syn*Ftn acts as a ‘transfer’ carboxylate with equivalent function to the conserved carboxylates of the animal ferritins, two variants, E141A and E141D, were generated.

The previously reported properties of *Syn*Ftn variant E62A suggested that Glu62 is involved in release of oxidized iron from the FOC (rather than in uptake of Fe^2+^ substrate). Inspection of crystal structures revealed that residue Asp65 is in a favourable conformation to coordinate iron together with Glu62 (Fig. 1d) despite the absence of evidence of iron binding at this position in *Syn*Ftn crystal structures. Variant D65A was therefore constructed to determine the importance of this residue in *Syn*Ftn function. A combination of protein crystallography and absorbance monitored solution kinetics was employed to probe the effect of each of the point mutations E141A, E141D and D65A on iron binding and oxidation by *Syn*Ftn. The data demonstrate that both Glu141 and Asp65 function in guiding Fe^2+^ to the FOC. Furthermore, in the absence of Asp65, the pathways for Fe^2+^ entry and Fe^3+^ exit to/from the FOC appear to clash.

## Methods

### Protein expression and purification

Plasmid DNA containing the sequence coding for wild-type *Syn*Ftn cloned into pET21b (Novagen) was provided by Professor Brian Palenik (Scripps Institution of Oceanography, UC San Diego). The sequences coding for variants D65A, E141A and E141D *Syn*Ftn cloned into pET21a (Novagen) were purchased from Genscript (Leiden, The Netherlands). The encoded proteins were overexpressed in *E. coli* strain BL21 DE3 (Promega). Cultures were grown at 37 °C with 200 r.p.m. shaking in Lysogeny Broth (LB) containing 100 μg ml^−1^ sodium ampicilin (Formedium) until the optical density at 600 nm was in the range 0.6–0.8. Protein expression was induced by the addition of isopropyl β-D-1 thiogalactopyranoside (IPTG) to a final concentration of 50 µm and cultures grown for a further 3 h at 37 °C, 200 r.p.m. shaking prior to harvesting by centrifugation. Cells were re-suspended in 20 mm HEPES pH 7.8 containing 100 mm KCl, 0.1 mm EDTA (buffer A), disrupted by sonication and debris removed by centrifugation at 40 000 ***g*** for 45 min. Thermally unstable proteins were precipitated from the supernatant by heating to 65 °C for 15 mins and removed by a further round of centrifugation (40 000 ***g*** for 45 min). Protein was precipitated from the supernatant via the addition of ammonium sulphate to a concentration of 0.55 g ml^−1^. Precipitated protein was pelleted by centrifugation at 40 000 ***g*** for 45 min, solubilized in the minimum volume of buffer A, and dialyzed against 1 l of identical buffer for 12 h. Contaminating proteins were removed by size exclusion chromatography (HiPrep 26/60 Sephacryl S-300HR, Cytiva) and contaminating DNA by anion exchange chromatography (HiTrap Q FF, Cytiva). For the latter, protein solutions were loaded in buffer A and eluted by stepping to 50 % buffer B (20 mm HEPES pH 7.8 containing 100 mm KCl, 1.0 m NaCl, 0.1 mm EDTA). Protein as isolated contained small quantities of iron that were removed according to the method of Bauminger *et al.* [34]. Following iron removal, protein was exchanged into 100 mm MES pH 6.5 by centrifugation over a 10 kDa molecular weight cut-off cellulose membrane (Millipore). Sample purity was assessed using SDS-PAGE and proteins judged to be free of DNA contamination once the ratio of absorbance at 277 nm and 260 nm reached 1.5.

### Protein crystallization and structure determination

Protein (10 mg ml^−1^) exchanged into 20 mm MES pH 6.5 in 2 µl drops was mixed with an equal volume of well solution (0.1 m sodium acetate, 2.0 m sodium chloride pH 4.6) and equilibrated in sitting drops by vapour diffusion against 200 µl of the same well solution. Crystals of bi-pyramidal morphology appeared within 24 h and grew to optimum size (100–150 µm) in approximately 1 week. Apo crystals were transferred to cryo-protectant comprising the well solution with pH adjusted to 6.5 and sodium chloride concentration increased to 2.2 m containing 30 % (v/v) glycerol prior to flash freezing in liquid nitrogen. Iron-enriched crystals were prepared by soaking for either 2 or 20 mins in well solution containing Fe^2+^ ions at 5 mm concentration and pH adjusted to 6.5. Crystals were then cryo-protected and frozen as above but using a solution containing 5 mm Fe^2+^ in addition to 30 % (v/v) glycerol. Diffraction data was collected at the Diamond Light Source using beamline i03 using wavelength 0.9795 Å for high-resolution data. Additional, highly redundant anomalous scattering data was collected from similarly treated iron-containing crystals at wavelengths corresponding to the peak of the iron K-edge (around 1.7399 Å). All data were indexed and processed using XDS and Aimless as part of the automatic xia2 pipeline [35]. Reprocessing was carried out as necessary using Aimless as part of the CCP4 programme suite [36]. Statistics are summarized in Table S1 (available in the online version of this article) for x-ray data used for structure solution and refinement and in Table S2 for data used for calculation of Bijvoet-difference Fourier (anomalous-scattering-density) maps.

Structure solution was performed by molecular replacement using phenix.phaser MR [37] with the 2.05 Å resolution structure of wild type *Syn*Ftn, pdb entry 5OUW [33], as the search model. In all cases the asymmetric unit contained a single copy of the protein monomer. Placement of metal ions was confirmed by reference to Bijvoet-difference Fourier maps (Fig. S1) calculated from anomalous scattering data (Table S2). Model refinement employed iterative cycles using phenix.refine [37] and manual correction using COOT [38]. No metal coordination restraints were applied to metal sites during refinement of iron-containing structures. Anisotropic temperature factor refinement was employed for all metal ions and their occupancies were manually adjusted to ensure that the average *B* factor of the metal fell within ±14 % of the B factors of atoms of their environment. The coordination geometry of metal binding sites was analysed after refinement using the CheckMyMetal web server [39]. Statistics relating to the metal binding sites in the refined structures can be found in Table S3.

### Kinetic analysis of iron oxidation and mineralization

Rates of Fe^2+^ oxidation were deduced from the rate of increase in absorbance at 340 nm due to the resulting ferric-oxo species, be they iron bound at the ferroxidase centre or in the mineral core. Assays employed 0.5 µm *Syn*Ftn in 100 mm MES pH 6.5 at 25 °C. Aerobic oxidation of ferroxidase centre bound Fe^2+^ following addition to apo wild-type *Syn*Ftn was complete in approximately 20 s. Accordingly the ability of variants D65A, E141A and E141D to support similar chemistry was monitored using stopped-flow absorbance spectroscopy. Fe^2+^ of the appropriate concentration in 1 mm HCl was mixed with 1 µm protein in 100 mm MES pH6.5 using an Applied Photophysics Bio-Sequential DX.17MV spectrophotometer with a 1 cm path length observation cell. The time dependences of absorbance increases at 340 nm were fitted to the sum of two exponential processes, encompassing rapid (r) and slow (s) components, using OriginPro 8 (OriginLab):

(1) $$\Delta A_{340}\left(t\right)=\Delta A_{340}^{\left(tot\right)}-\Delta A_{340}^{r}e^{-k_{r}t}-\Delta A_{340}^{s}e^{-k_{s}t}$$

The extent to which oxidized iron vacates the ferroxidase centres was investigated by monitoring the regeneration of the rapid phase of iron oxidation associated with the apo protein. 1 µm protein was incubated with 200 µm Fe^2+^ at 25 °C until the absorbance at 340 nm became invariant with time. Equivalent samples were then mixed with an equal volume of 72 µm Fe^2+^ in 1 mm HCl either immediately or following a further period of incubation of 3, 8, 15 or 60 mins. An equivalent sample was incubated at 25 °C for 60 mins followed by a further 15 h at 4 °C. After re-equilibration at 25 ˚C the protein was mixed with an equal volume of 72 µm Fe^2+^ in 1 mm HCl as above.

Assays to assess the rate at which the proteins were able to mineralize iron within the internal cavity employed a higher iron to protein stoichiometry of 400 Fe^2+^ per *Syn*Ftn. Time dependence of the 340 nm absorbance was recorded on a Hitachi U-2900 spectrometer with the sample chamber maintained at 25 °C following manual mixing of 6.4 µl of a 50 mm Fe^2+^ solution in 50 mm HCl to a 1.6 ml sample of 0.5 µm protein in 100 mm MES pH 6.5. The increased ratio of Fe^2+^:protein resulted in dependencies that were approximately linear for the initial 180 s of the reaction. Initial rates of iron mineralization were deduced from the gradient of this linear region and an extinction coefficient for the mineral core calculated from the net absorbance change upon complete oxidation of the 200 µm Fe^2+^ added.

### Electron paramagnetic resonance

In total, 4.17 µm (100 µm in monomer) *Syn*Ftn in 100 mm MES pH 6.5 was manually mixed with 72 equivalents of Fe^2+^ in a syringe needle before expelling into a quartz tube and flash frozen in methanol cooled by solid CO_2_ approximately 10 s after mixing. Spectra were recorded on a Bruker EMX spectrometer equipped with an Oxford Instruments liquid-helium flow cryostat using the following parameters: temperature 10 K, modulation frequency 100 kHz, modulation amplitude 5 G, time constant 82 ms, scan rate 22.6 G s^−1^ and microwave power 3.19 mW.

## Results

### *Syn*Ftn Glu141 is involved in transfer of Fe^2+^ from the threefold channel to the FOC

**Fig. 2.**
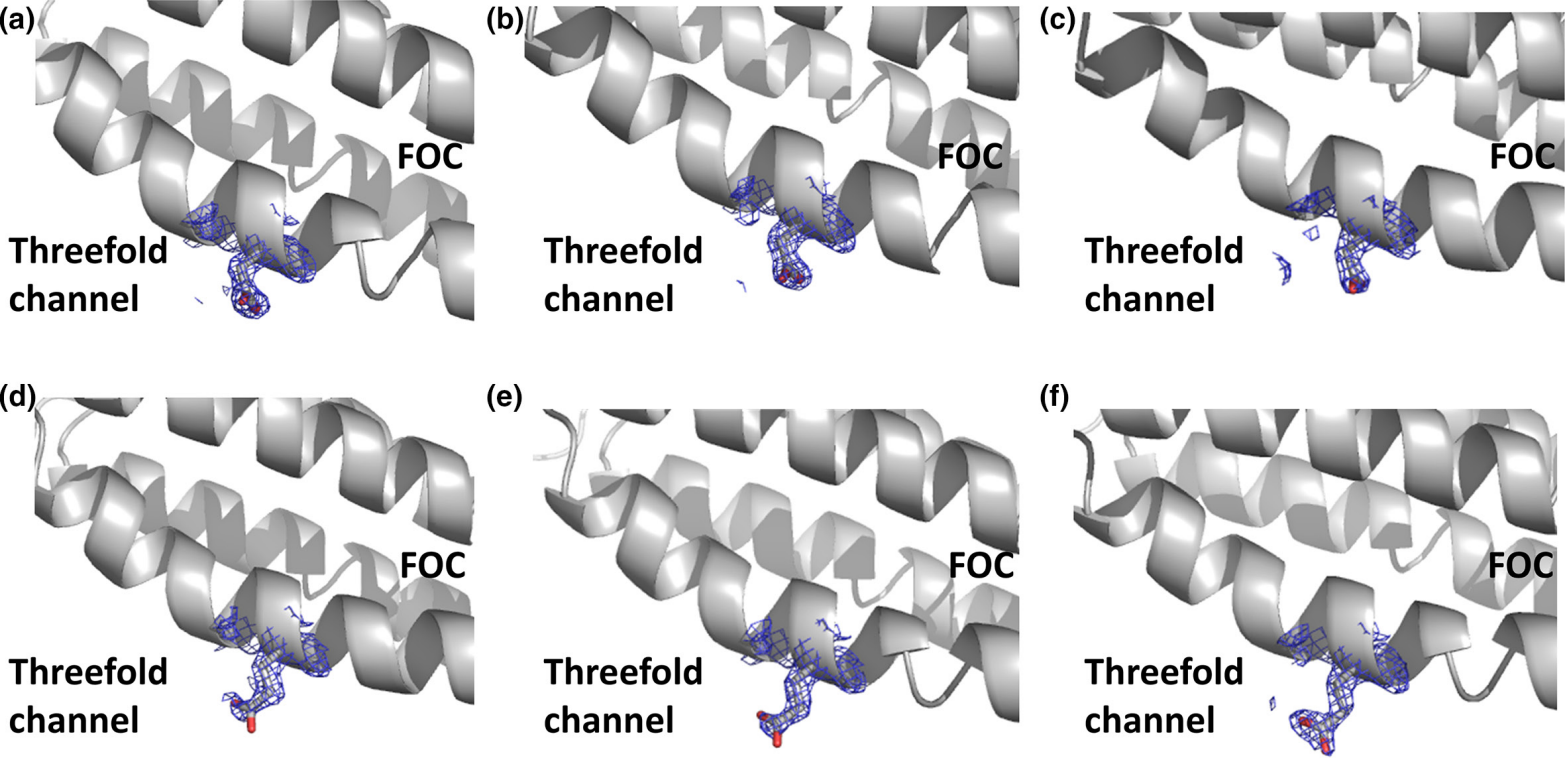
Orientation of residue 141 in SynFtn variants. Double difference Fourier (2mF_o_-DF_c_) map within 4 Å of the beta carbon atom of residue 141 contoured at 1.5 σ represented as a blue mesh, and the side chain of residue 141 shown in stick representation. In the metal-free (a), 2 min iron-soaked (b) and 20 min iron-soaked (c) structures of variant E141D, Asp141 is oriented toward the FOC. In contrast in the metal-free (d), 2 min iron-soaked (e) and 20 min iron-soaked (f) structures of variant D65A, the side chain of Glu141 is oriented toward the threefold channel.

Variants E141A and E141D both crystallized under conditions identical to those reported for wild-type *Syn*Ftn [33]. The wild-type protein contains three iron binding sites: high- and low-affinity binding sites at the FOC, sites 1 and 2, respectively, and site 3 located in the threefold channel and coordinated by the side chains of three symmetry equivalent Asp137 residues. Protein structures derived from all crystals harvested without iron enrichment were free of bound metal ions, as expected (Table S3). The structures of variants E141A and E141D overlay with that reported for the wild-type protein with overall rmsd (for main chain atoms) of 0.10 and 0.11 Å, respectively, with the major difference in structure being associated with the side chain of the substituted residue. Typically when site 3 is vacant, residue 141 is oriented toward the FOC. This is the case for the metal-free structure of variant E141D (Fig. 2), while conformational flexibility at this position is not possible in E141A. In contrast to the behaviour of wild-type *Syn*Ftn, exposure of E141D variant crystals to Fe^2+^ for either 2 or 20 min did not lead to a change in conformation of the residue at position 141, regardless of the fractional occupancy of site 3 (Fig. 2).

In wild-type *Syn*Ftn, increasing the length of iron exposure resulted in a reduction of the distance between the iron ions located at sites 1 and 2 from 3.90 to 3.46 Å and this was interpreted as reflecting the change in oxidation state from mixed valent Fe^2+^/Fe^3+^ to di-Fe^3+^ [33]. Variant E141D showed little change in metal ion position or occupancy between long and short soaking times, with both resembling the 20 min iron-soaked structure of wild-type but with lower occupancy of site 3 (Fig. S3). The apparent absence of a mixed valent site at short soak time may reflect a destabilization of this form relative to the di-Fe^3+^ form, at least in the crystal. Overall, though, the substitution E141D appeared to have little effect on the iron uptake properties of the protein, despite Asp141 being oriented toward the FOC in all structures, suggesting a loss in conformational flexibility in the side chain of this residue.

In contrast, the fractional occupancy of all metal binding sites in the structures of variant E141A was lower than the equivalent soak for the wild-type protein, suggesting that substitution of Glu141 by an aliphatic residue results in impaired uptake of Fe^2+^ substrate (Fig. S4). Although increasing the soaking time led to an apparent reduction in the separation between iron at sites 1 and 2 from 3.69 to 3.61 Å, such a small change is unlikely to reflect a change in oxidation state from a mixed valent to di-ferric FOC. This would be consistent with a lower rate of oxidation catalysed by the E141A variant.

To probe whether the iron binding characteristics observed in the iron-enriched structures of the Glu141 variants were linked to changes in iron uptake activity, stopped-flow kinetic methods were employed. Wild-type *Syn*Ftn exhibits a multiphasic oxidation of iron within the first several seconds of aerobic mixing of metal-free protein with solutions of Fe^2+^. The rate of the most rapid of these phases is linearly dependent on the concentration of Fe^2+^, indicating that the binding of iron is rate-limiting for its oxidation. Performing assays of Fe^2+^ oxidation activity under pseudo first-order conditions allows the second-order rate constant for the binding of iron to be determined from the gradient of a plot of apparent first order rate constant vs. concentration of Fe^2+^. For wild-type *Syn*Ftn, this analysis yielded a rate constant of 3.7×10^5^ m ^−1^ s^−1^ under the conditions reported [33]. Disruption by site-directed mutagenesis of transient binding sites involved in the transport of Fe^2+^ to the FOC would be expected to result in a decrease in this rate constant for the variant proteins. Equivalent measurements to the iron oxidation assays performed with wild-type *Syn*Ftn were therefore performed with the Glu141 variants to determine its importance for function.

**Fig. 3.**
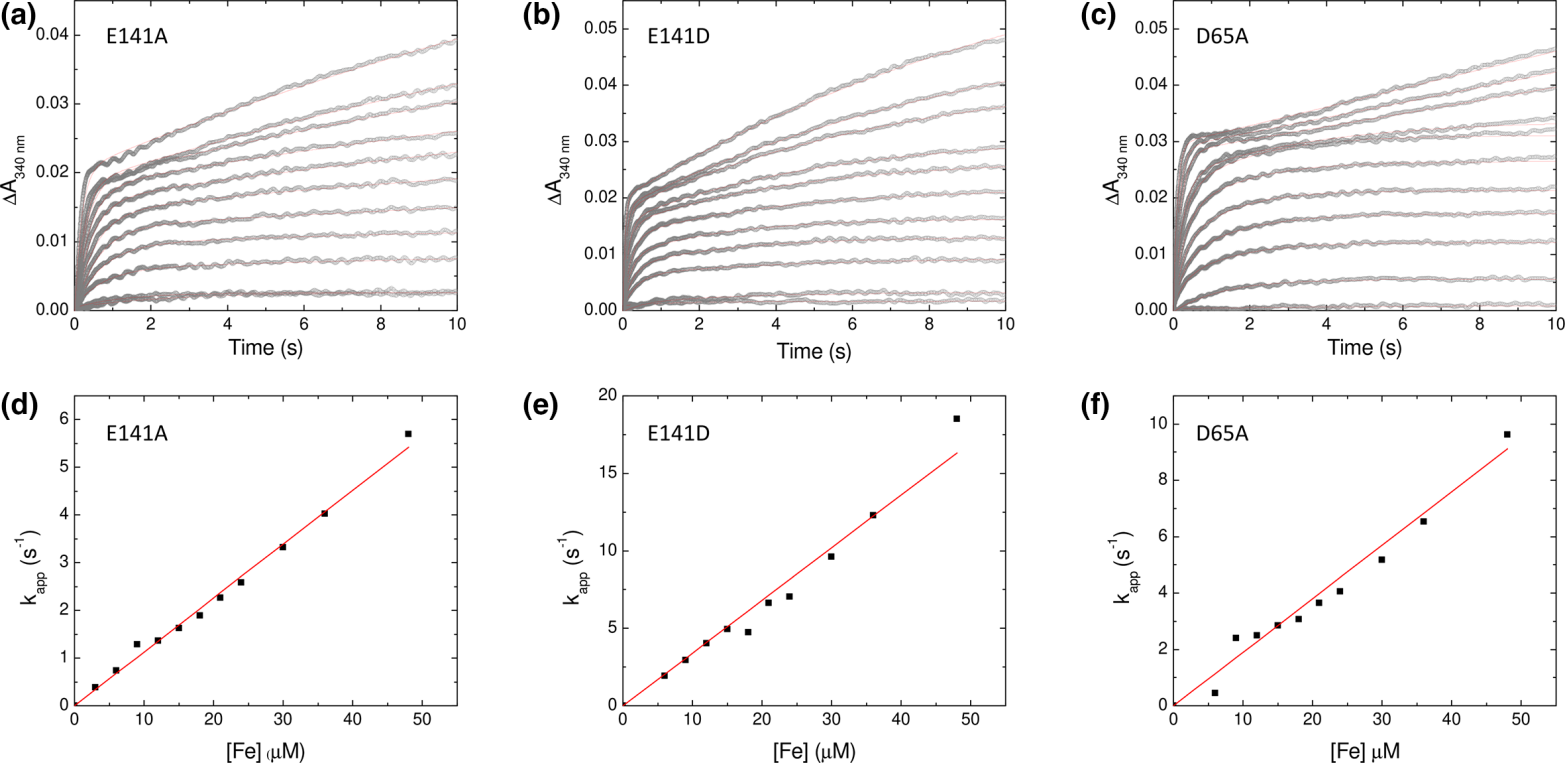
Rapid Fe^2+^ oxidation catalysed by SynFtn variants. The change in absorbance at 340 nm in the first 10 s following the aerobic mixing of equal volumes of Fe^2+^ solutions (6 to 96 µm in 1 mm HCl) with 1 µm solutions of SynFtn variant E141A (a) E141D (b) or D65A (c) in 100 mm MES pH 6.5. The linear dependence of the apparent first order rate constant for Fe^2+^ oxidation as a function of [Fe^2+^] is plotted for variant E141A (d), E141D (e) and D65A (f).

The increase in absorbance at 340 nm as a function of time at each of the Fe^2+^ concentrations employed together with the dependence of the apparent first order rate constants on Fe^2+^ concentration for each of the Glu141 variant proteins are shown in Fig. 3. Both E141A and E141D exhibited a biphasic response with the rate of the rapid phase linearly dependent on Fe^2+^ concentration. In the case of E141A the second-order rate constant extracted from the gradient of this plot was 1.1×10^5^ m ^−1^ s^−1^, significantly lower than that measured for wild-type *Syn*Ftn. In contrast, the rate constant for iron binding to the E141D variant was almost identical to the wild-type protein, at 3.4×10^5^ m ^−1^ s^−1^, demonstrating that the conservative replacement of Glu by Asp did not have a significant effect on oxidation rate. Thus, the absence of observed conformational flexibility of residue D141 in the crystal structures is not associated with a reduction in the rate of iron uptake. The presence or absence of a negatively charged side chain appears to have a greater functional impact.

### *Syn*Ftn Asp65 is also involved in transfer of Fe^2+^ from the threefold channel to the FOC

Variant D65A also crystallized under identical conditions to those reported for wild-type *Syn*Ftn [33], and the resulting D65A structure overlays with that for the wild-type protein (overall rmsd of 0.09 Å). In the metal-free structure of variant D65A the side chain of Glu141 is oriented towards the threefold channel (Fig. 2), and, in contrast to wild-type protein, the exposure of crystals to Fe^2+^ for either 2 or 20 min did not lead to a change in conformation of Glu141, regardless of the fractional occupancy of site 3.

The structures of iron-exposed variant D65A show the same general trend in metal ion occupancy and position as reported for wild-type protein (Table S3). Increasing the soaking time led to an increase in the fractional occupancy of each of the iron binding sites and also a decrease in the separation between irons bound at sites 1 and 2, albeit a less significant change from 3.70 to 3.55 Å (Fig. S2). The most significant differences between wild-type and D65A *Syn*Ftn structures related to a biassing of FOC occupancy towards the high affinity site 1 and occupancy of site 3, even at the shorter soaking time.

The capacity of the D65A variant to catalyse Fe^2+^ oxidation and core mineralization was investigated. The response of variant D65A was distinct from that of either wild-type *Syn*Ftn or any variant reported to date. Whilst the increase in 340 nm absorbance was biphasic, the rapid phase saturated at an absorbance change approximately double that of other *Syn*Ftn proteins. This was followed by a brief plateau before a much slower phase of iron oxidation that likely corresponded to the onset of formation of the mineral core, rather than oxidation of a mixed valent FOC, as is typically observed for *Syn*Ftn. However, electron paramagnetic resonance (EPR) spectroscopy confirmed the formation of a Fe^2+^/Fe^3+^ mixed valent intermediate during iron oxidation at the FOC of variant D65A (Fig. S5). This most likely arises from changes in the relative rates of formation and loss of the mixed valent FOC. Fitting of the increase in 340 nm absorbance in the first second following mixing demonstrated that rate of the rapid phase is also linearly dependent on iron concentration for this protein. The second-order rate constant extracted from these data is 1.9×10^5^ m ^−1^ s^−1^. The rate of iron binding to *Syn*Ftn FOCs is therefore impaired by the D65A substitution, but to a lesser extent than that observed for E141A.

### Substitution of Asp65 indirectly affects release of Fe^3+^ from the *Syn*Ftn FOC

**Fig. 4.**
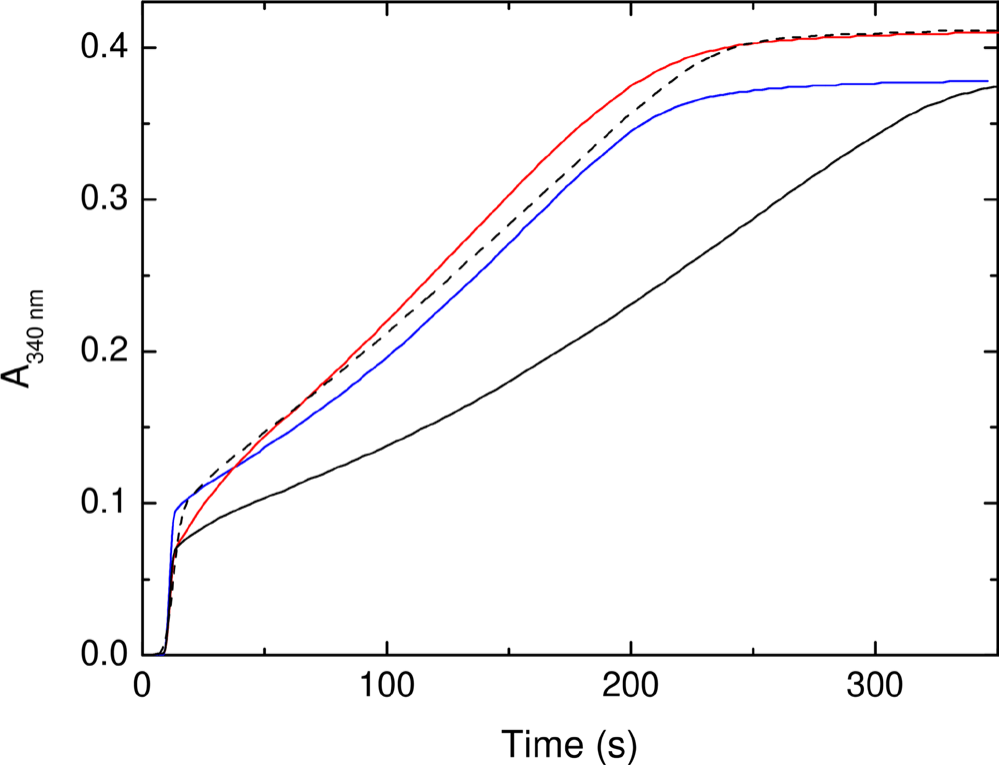
Iron mineralization by SynFtn variants. Increase in 340 nm absorbance as a function of time following addition of 400 equivalents of Fe^2+^ to 0.5 µm D65A (black trace), E141A (red trace) and E141D (blue trace) SynFtn in 100 mm MES pH 6.5. The broken black trace shows the response of the wild-type protein for comparison.

*Syn*Ftn is thought to lay down a mineral core in the internal cavity of the protein via a classic ‘displacement’ mechanism. Hydrolysis of the metastable di-Fe^3+^ form of the FOC results in the dissociation of iron from the catalytic centre, regenerating vacant sites for multiple turnover and initiating mineral core formation at as yet undefined nucleation sites. In the wild-type protein release of iron from FOC sites is rate-limiting for this process, meaning the rate of mineral core formation can be correlated with the efficiency of displacement of Fe^3+^ from the FOC. Fig. 4 shows the increase in absorbance at 340 nm following the addition of 400 equivalents of Fe^2+^ to each of the variant proteins, sufficient to lead to an average of eight turnovers per FOC. Variants E141A and E141D are both able to mineralize iron at almost the same rate (~80 %) as determined for the wild-type protein, suggesting that release of oxidized product from FOC sites is not significantly impaired by substitution of Glu141. In contrast, the initial rate of mineral core formation in variant D65A is 0.35 µm s^−1^, only ~35 % of the 0.95 µm s^−1^ rate of wild-type *Syn*Ftn, consistent with the hypothesis that Asp65 plays a role in transporting ferric-oxo species away from the FOC.

It has been postulated that ferritins that operate via the displacement mechanism for mineral core formation require incoming Fe^2+^ to trigger release of oxidized iron from FOC sites [40]. However, following multiple turnovers of the FOC, the rapid Fe^2+^ oxidation activity of wild-type *Syn*Ftn regenerates over a period of 15–60 min [33]. Rapid oxidation occurs on a timescale at least an order of magnitude shorter than that of Fe^3+^ displacement, and, therefore, requires vacant FOC sites in order to be observed. This means that Fe^3+^ must spontaneously exit the FOC in the wild-type protein. The timescale of this process compared to the rate of *Syn*Ftn-catalysed iron mineralization clearly indicates that Fe^3+^ release from the FOC is accelerated by incoming Fe^2+^. Therefore, the observed rates of mineralization for the *Syn*Ftn variant proteins may be influenced by both the stability of the di-Fe^3+^ form of the FOC, and the differing rates at which incoming Fe^2+^ accesses the catalytic centre.

**Fig. 5.**
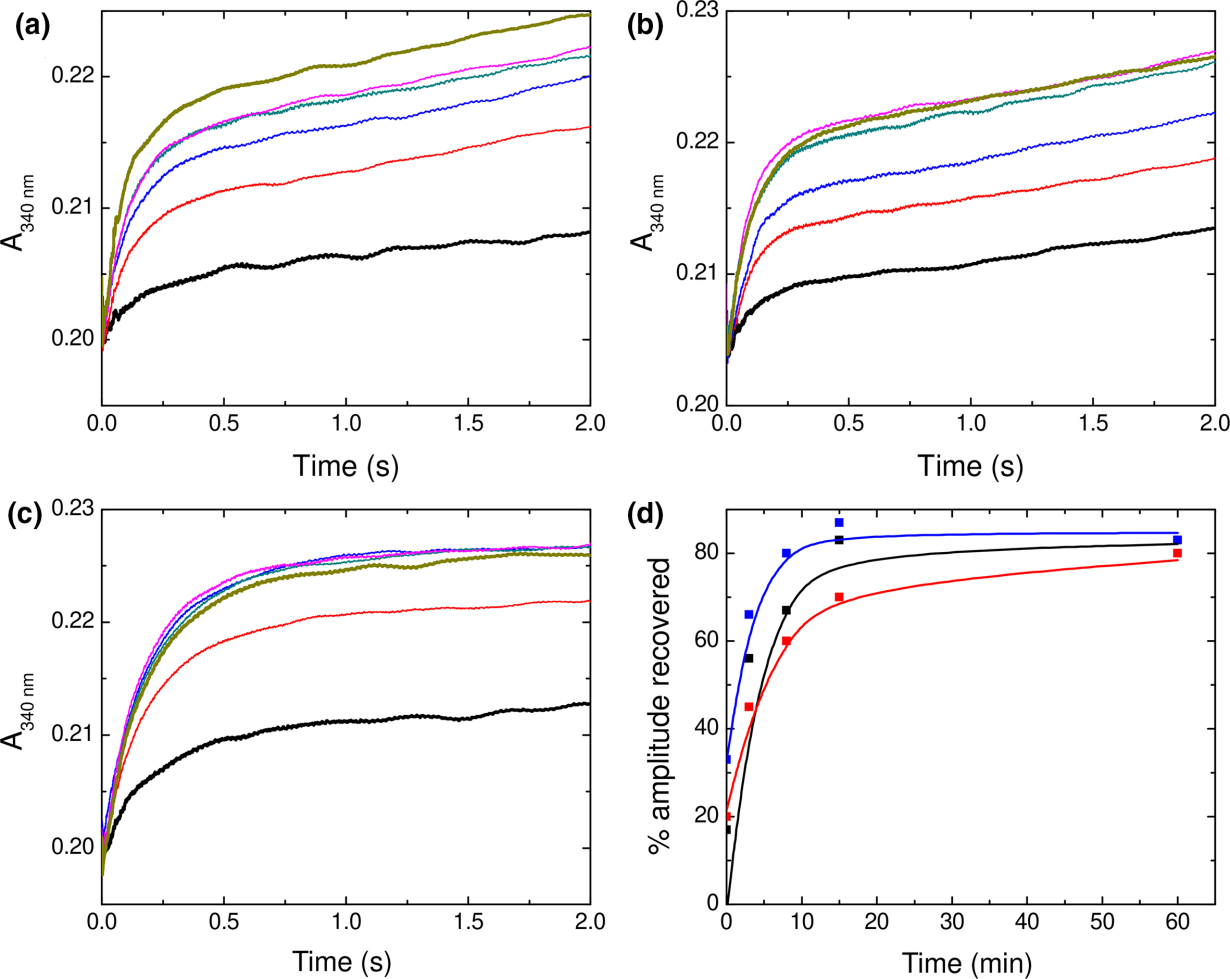
Regeneration of rapid iron oxidation by SynFtn. Increase in absorbance at 340 nm as a function of time following the mixing of (a) wild-type, (b) E141D and (c) D65A SynFtn with 72 equivalents of Fe^2+^ at pH 6.5, 25 ˚C immediately (black trace), 3 min (red trace), 8 min (blue trace), 15 min (dark teal trace), 60 min (magenta trace) and 16 h (dark yellow trace) following the complete oxidation of 200 equivalents of Fe^2+^. (d) The extent to which the rapid oxidation phase observed for metal-free protein is recovered as a function of time for wild-type (black data points), E141D (red data points) and D65A (blue data points) SynFtn. Solid traces represent fits to mono-exponential functions with decay constants of 4, 6.5 and 3 min, respectively.

The rate and extent to which rapid activity regenerates following multiple FOC turnovers can be used to probe the relative stability of the di-Fe^3+^ FOC in the variant proteins in comparison to wild-type *Syn*Ftn to establish the importance of this for the rate at which the mineral core is formed. The onset of rapid Fe^2+^ oxidation following the mineralization of 200 equivalents of iron is shown for wild-type and E141D *Syn*Ftn in Fig. 5. The slower rate of iron binding to variant E141A precluded accurate deconvolution of rapid and slow phases for the small amplitudes of the former at early time points. The iron release properties of E141D were almost identical to wild-type *Syn*Ftn, consistent with the above demonstration that Glu141 plays no significant role in the transfer of Fe^3+^ out of the FOCs. Surprisingly, however, the rate at which the rapid iron oxidation phase of the apo protein was regenerated in variant D65A was also almost identical to that of the wild-type protein. Therefore, the impaired mineralization activity of variant D65A cannot be a consequence of increased stability of the di-Fe^3+^ form of the FOC, as was reported for variant E62A [18], suggesting that the replacement of Asp65 has a less direct effect on the exit of Fe^3+^ from the FOC.

## Discussion

*Syn*Ftn has been demonstrated to take up Fe^2+^ via the threefold channel [18], the same route utilized by animal ferritins [23]. However, the conserved glutamate residues required for transport of substrate from the inner surface exit of this channel to the FOC of animal ferritins are absent from the sequence of *Syn*Ftn. Furthermore, transient iron binding sites have not been crystallographically defined in *Syn*Ftn, possibly as a consequence of the different conditions under which the protein crystallizes. Two sites on the inner surface of *Syn*Ftn were identified as potentially important for the transport of iron through the protein and substitutions introduced at positions 65 and 141 in the peptide sequence with the aim of disrupting the contributions made by these residues in the wild-type protein.

The data reported here demonstrates that transport of iron to the FOC is inhibited in *Syn*Ftn variant E141A. However, the effect is less severe than that caused by disruption of the threefold channel by the D137A substitution that led to loss of all rapid Fe^2+^ oxidation activity [18]. This suggests that residue Glu141 is not essential for Fe^2+^ to traverse the protein coat, and that its role is to direct incoming metal toward the catalytic FOCs. Consistent with this, variant E141D was able to bind, oxidize and mineralize iron all at similar rates to the wild-type protein, despite the lack of any crystallographic evidence for conformational flexibility of Asp141. It appears, therefore, that the charge of the carboxylate group is of greater importance for *Syn*Ftn function than the observed conformational flexibility of residue Glu141, despite the altered fractional occupancy of *Syn*Ftn iron binding sites induced by the E141D substitution. Given its location between the threefold channel and the FOC, it is unsurprising that Glu141 does not play a role in the transfer of Fe^3+^ out of the FOC into the protein’s cavity.

High-resolution structures of *Syn*Ftn revealed that the side chain of residue Asp65 lies in close proximity to Glu62, raising the possibility that the two residues form a transient binding site for iron. Given the reported properties of variant E62A and its importance for the transfer of Fe^3+^ out of the FOC, we predicted that D65A *Syn*Ftn would be inhibited in its ability to mineralize iron due to increased stability of the di-Fe^3+^ form of the FOC. However, the data presented here suggest that the roles of residues Glu62 and Asp65 differ significantly.

Firstly, substitution of Glu62 led to a reduction in the rate of rapid iron oxidation such that iron binding was no longer rate-limiting for this process [18]. No such effect was observed on substitution of Asp65. However, the rate at which Fe^2+^ binds to FOC sites in variant D65A was only 50 % that of the wild-type protein under the same conditions, suggesting a role for Asp65 in uptake of Fe^2+^. In contrast there was no evidence that Fe^2+^ binding to the FOC was inhibited by the substitution E62A. Whilst variant D65A was inhibited to almost the same extent as E62A in its ability to form a mineral core, the rate at which rapid FOC activity returned following multiple turnovers was close to that of the wild-type protein, demonstrating that slower mineralization is not due to increased stability of the di-Fe^3+^ site. Therefore, it appears that, in variant D65A, the release of Fe^3+^ from the FOC is only inhibited in the presence of incoming Fe^2+^ substrate. Given that Fe^2+^ uptake is inhibited in this protein, it appears that substitution of residue Asp65 alters the pathway of Fe^2+^ entry to the FOC, such that the substrate blocks the route of Fe^3+^ exit from the FOC, thereby inhibiting the rate of mineral formation.

**Fig. 6.**
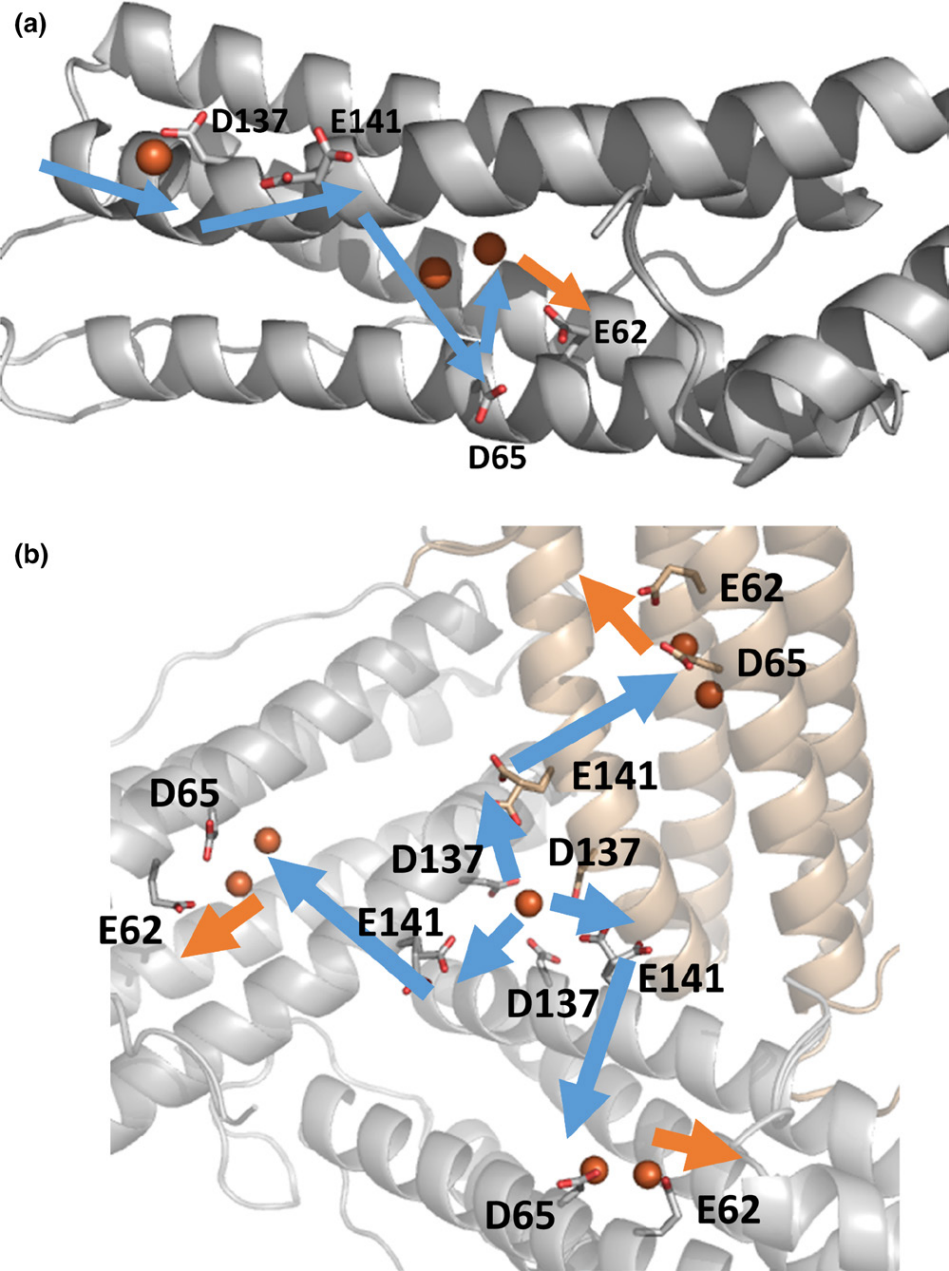
Proposed route of iron transport through SynFtn as viewed from the inner surface of the protein. Pale blue arrows represent the route of Fe^2+^ into the FOC, while the orange arrow indicates the exit of Fe^3+^ following oxidation. Electrostatic interaction with the carboxylate group of Asp137 leads to Fe^2+^ uptake via the threefold channel. Once Fe^2+^ reaches the inner surface of the protein it is directed toward the FOC by Glu141. The sidechain of Asp65 sits below site 2, the low affinity Fe binding site of the FOC, and facilitates iron binding at the catalytic centre. Following oxidation in the FOC the product exits via coordination to the side chain of residue Glu62. (a) Viewed along the inner surface of a single monomer. (b) Viewed from the inner surface of the threefold channel.

The 24meric ferritins do not appear to be widely distributed among cyanobacteria. Encoding of several ‘mini ferritins’, each composed of 12 identical protomers and with distinct physiological roles, is seemingly more common [41]. However, over 100 homologues of *Syn*Ftn have been identified and these are confined almost exclusively to the marine picocyanobacteria *Synechococcus* and *Prochlorococcus *. As such, *Syn*Ftn may represent the first characterized example of a new class of ferritins unique to the cyanobacteria. Whilst it shares a common route of Fe^2+^ entry with animal ferritins, via the threefold channel, there is very little sequence similarity between the channels of the two classes of ferritin. The more stringent requirement of the animal ferritins that the channel contains a Cys at the entrance on the outer surface together with two rings of carboxylates within the channel may reflect the fact that these proteins are heteropolymers composed of both H- and l-chains and the substrate should be directed only to the H-chains as these harbour the FOC sites. *Syn*Ftn is a homopolymer of H-chain like units and, as such, incoming substrate can be directed to any of the three monomers bordering each of the threefold channels since they all contain a catalytic centre for its oxidation. This appears to place less stringent requirements on the composition of the threefold channel with only a single carboxylate residue at the mid-channel position. As a consequence, *Syn*Ftn employs a distinct route of iron transit through the protein. After crossing the protein coat via the threefold channel, Fe^2+^ is shuttled to the FOC via Glu141 and then Asp65. These may constitute part of transient binding sites equivalent to site 4 and site 3 of the animal ferritins, respectively. Other residues involved in any such sites are yet to be identified. Following binding to, and oxidation at, the FOC iron exits via Glu62, initiating the process of mineralization. The proposed route of iron through wild-type *Syn*Ftn is summarized in Fig. 6. In the absence of Asp65, the pathway for Fe^2+^ into the FOC may be altered, apparently resulting in inhibition of Fe^3+^ egress, and suggesting that the pathways for iron entry and exit from the FOC are closely associated, such that disruption of the uptake route can cause a clash with the exit route.

In contrast to animals, in which the majority of tissues produce only one type of ferritin, it is common for the genomes of prokaryotes to encode multiple ferritin genes, often expressed in response to different environmental cues. Examples isolated from different prokaryotic organisms exhibit a far greater range of variation in the rates of iron binding, oxidation and product release than their animal counterparts, possibly reflecting the diversity of environmental niches inhabited by unicellular organisms and the need to adapt rapidly to changes in the extracellular environment. The data presented here provides evidence of variation in the routes of iron uptake and release employed in prokaryotic ferritins, which may underpin the reported variation in the kinetics of these processes and, therefore, the ability of microbes to adapt rapidly to oxidative stress or changes in iron availability.

## Supplementary Data

Supplementary material 1
