# Supplementary material for: Key carboxylate residues for iron transit through the prokaryotic ferritin SynFtn
Source: Microbiology (Reading). 2021 Nov 26;167(11):001105. doi: 10.1099/mic.0.001105 (PMC8743623; doi:10.1099/mic.0.001105)
Supplement: Supplementary material 1 [file mic-167-1105-s001.pdf]

Supplementary Material

Key carboxylate residues for iron transit through the prokaryotic ferritin *Syn*Ftn

Justin M. Bradley[1], Joshua Fair[1], Andrew M. Hemmings[1] and Nick E. Le Brun[1*]

[1]Centre for Molecular and Structural Biochemistry, School of Chemistry, University of East Anglia, Norwich, NR4 7TJ, UK.
[2]Centre for Molecular and Structural Biochemistry, School of Biological Sciences, University of East Anglia, Norwich, NR4 7TJ, UK.

**PDB code**	7PF8	7PFG	7PFJ
Fe^{2+} soak (min)	0	2	20
Wavelength (Å)	0.9763	0.9763	0.9763
Resolution range (Å)	39.54 - 1.85 (1.916 - 1.85)	39.52 - 1.8 (1.865 - 1.8)	40.53 - 1.65 (1.709 - 1.65)
Space group	F 4 3 2	F 4 3 2	F 4 3 2
Unit Cell	176.85 176.85 176.85<br>90 90 90	176.75 176.75 176.75<br>90 90 90	176.67 176.67 176.67<br>90 90 90
Total reflections	41592 (4034)	45020 (4411)	57966 (5664)
Unique reflections	20800 (2018)	22511 (2206)	28983 (2832)
Multiplicity	2.0 (2.0)	2.0 (2.0)	2.0 (2.0)
Completeness (%)	100 (100)	100 (100)	100 (100)
Mean I/sigma(I)	40.00 (2.45)	53.82 (3.03)	48.96 (3.40)
Wilson B-factor	39.08	37.05	29.92
R-merge	0.008425 (0.2284)	0.006702 (0.2062)	0.00785 (0.1877)
R-meas	0.01191 (0.323)	0.009478 (0.2916)	0.0111 (0.2655)
CC 1/2	1 (0.948)	1 (0.917)	1 (0.921)
CC*	1 (0.987)	1 (0.978)	1 (0.979)
Reflections used in refinement	20797 (2018)	22510 (2206)	28982 (2832)
Reflections used in R-free	1006 (86)	1142 (109)	1412 (133)
R-work	0.1933 (0.3203)	0.1767 (0.2564)	0.1511 (0.1548)
R-free	0.2172 (0.4012)	0.2009 (0.3330)	0.1768 (0.2272)
CC(work)	0.963 (0.864)	0.967 (0.872)	0.968 (0.952)
CC(free)	0.957 (0.719)	0.966 (0.681)	0.951 (0.872)
No. of non-hydrogen atoms	1587	1616	1643
Macromolecules	1406	1403	1409
Ligands	6	4	11
Solvent	175	209	223
Protein residues	178	178	178
RMS bonds	0.007	0.006	0.005
RMS angles	0.71	0.68	0.69
Ramachandran favoured (%)	99	99	100
Ramachandran allowed (%)	0.56	0.56	0
Ramachandran outliers (%)	0	0	0
Rotamer outliers (%)	2	0.67	0.66
Clashscore	1.44	0.00	1.80
Average B-factor	34.67	33.81	31.04
Macromolecules	33.63	32.54	29.02
Ligands	48.97	35.02	44.72
Solvent	42.60	42.26	43.14

Table S1 Data collection and refinement statistics for *Syn*Ftn E141A collected at 0.97 Å. Statistics for the highest resolution bin are shown in parentheses.

**PDB code**	7PF9	7PFH	7PFK
Fe^{2+} soak (min)	0	2	20
Wavelength (Å)	0.9763	0.9763	0.9763
Resolution range (Å)	40.52 - 1.55 (1.606 - 1.55)	44.22 - 1.5 (1.554 - 1.5)	40.51 - 1.55 (1.605 - 1.55)
Space group	F 4 3 2	F 4 3 2	F 4 3 2
Unit Cell	176.607 176.607 176.607 90 90 90	176.87 176.87 176.87 90 90 90	176.59 176.59 176.59 90 90 90
Total reflections	69252 (6570)	76744 (7495)	69324 (6708)
Unique reflections	34627 (3285)	38387 (3754)	34688 (3374)
Multiplicity	2.0 (2.0)	2.0 (2.0)	2.0 (2.0)
Completeness (%)	100 (96)	100 (99)	100 (99)
Mean I/sigma(I)	25.72 (2.15)	35.01 (2.26)	23.97 (1.54)
Wilson B-factor	26.38	26.26	28.45
R-merge	0.007727 (0.3076)	0.009064 (0.3128)	0.01462 (0.4388)
R-meas	0.01093 (0.435)	0.01282 (0.4423)	0.02067 (0.6205)
CC 1/2	1 (0.676)	1 (0.737)	0.999 (0.639)
CC*	1 (0.898)	1 (0.921)	1 (0.883)
Reflections used in refinement	34627 (3285)	38387 (3754)	34687 (3373)
Reflections used in R-free	1758 (165)	1810 (166)	1743 (164)
R-work	0.1595 (0.2649)	0.1533 (0.2042)	0.1532 (0.2252)
R-free	0.1804 (0.3082)	0.1718 (0.2371)	0.1865 (0.2673)
CC(work)	0.968 (0.775)	0.968 (0.885)	0.968 (0.825)
CC(free)	0.972 (0.672)	0.961 (0.879)	0.966 (0.755)
No. of non-hydrogen atoms	1607	1629	1628
Macromolecules	1413	1416	1410
Ligands	4	10	4
Solvent	190	203	214
Protein residues	178	178	178
RMS bonds	0.005	0.004	0.005
RMS angles	0.65	0.66	0.67
Ramachandran favoured (%)	100	100	100
Ramachandran allowed (%)	0	0	0
Ramachandran outliers (%)	0	0	0
Rotamer outliers (%)	0	1.3	2.6
Clashscore	1.08	3.22	1.44
Average B-factor	34.86	30.76	32.33
Macromolecules	33.04	28.94	30.52
Ligands	50.51	51.10	35.74
Solvent	48.13	42.44	44.17

Table S1 (continued) Data collection and refinement statistics for *Syn*Ftn E141D collected at 0.97 Å. Statistics for the highest resolution bin are shown in parentheses.

PDB code	7PF7	7PFB	7PFI
Fe^{2+} soak (min)	0	2	20
Wavelength (Å)	0.9763	0.9763	0.9763
Resolution range (Å)	40.64 - 1.7 (1.761 - 1.7)	40.66 - 1.7 (1.761 - 1.7)	40.47 - 1.7 (1.761 - 1.7)
Space group	F 4 3 2	F 4 3 2	F 4 3 2
Unit Cell	177.14 177.14 177.14<br>90 90 90	177.24 177.24 177.24<br>90 90 90	176.4 176.4 176.4<br>90 90 90
Total reflections	53527 (5228)	53623 (5269)	52870 (5178)
Unique reflections	26766 (2616)	26812 (2635)	26436 (2589)
Multiplicity	2.0 (2.0)	2.0 (2.0)	2.0 (2.0)
Completeness (%)	100 (100)	100 (100)	100 (100)
Mean I/sigma(I)	57.66 (3.11)	38.10 (2.53)	54.47 (3.29)
Wilson B-factor	32.99	32.23	32.04
R-merge	0.005482 (0.1981)	0.008573 (0.2489)	0.007985 (0.1879)
R-meas	0.007753 (0.2801)	0.01212 (0.352)	0.01129 (0.2658)
CC 1/2	1 (0.919)	1 (0.868)	1 (0.928)
CC*	1 (0.979)	1 (0.964)	1 (0.981)
Reflections used in refinement	26765 (2616)	26811 (2635)	26436 (2589)
Reflections used in R-free	1327 (129)	1341 (127)	1315 (124)
R-work	0.1817 (0.2715)	0.1738 (0.2616)	0.1731 (0.2507)
R-free	0.2076 (0.2706)	0.1989 (0.2954)	0.1935 (0.2807)
CC(work)	0.966 (0.841)	0.968 (0.843)	0.969 (0.867)
CC(free)	0.949 (0.799)	0.943 (0.729)	0.944 (0.816)
No. of non-hydrogen atoms	1640	1647	1644
Macromolecules	1424	1418	1410
Ligands	4	5	5
Solvent	212	224	229
Protein residues	178	178	178
RMS bonds	0.005	0.006	0.005
RMS angles	0.72	0.71	0.71
Ramachandran favoured (%)	100	100	100
Ramachandran allowed (%)	0	0	0
Ramachandran outliers (%)	0	0	0
Rotamer outliers (%)	0.66	0.66	0.66
Clashscore	1.07	1.43	1.80
Average B-factor	31.93	30.86	31.28
Macromolecules	30.47	29.32	29.59
Ligands	41.91	34.83	31.51
Solvent	41.56	40.54	41.66

Table S1 (continued) Data collection and refinement statistics for *Syn*Ftn D65A collected at 0.97 Å. Statistics for the highest resolution bin are shown in parentheses.

**Data collection**	**E141A 2 min. soak**	**E141A 20 min. soak**	**E141D 2 min. soak**	**E141D 20 min. soak**	**D65A 2 min. soak**	**D65A 20 min. soak**
Wavelength (Å)	1.7385	1.7385	1.7395	1.7395	1.7385	1.7385
Space group	F432	F432	F432	F432	F432	F432
Cell parameter (Å)	176.92	176.72	177.64	176.2	177.21	176.63
Resolution limits (Å)	62.55 - 2.2 (2.279 - 2.2)	62.48 - 2.2 (2.279 - 2.2)	51.28 - 2.202 (2.281 - 2.202)	53.13 - 2.2 (2.279 - 2.2)	53.43 - 2.2 (2.279 - 2.2)	53.26 - 2.2 (2.279 - 2.2)
No. unique reflections	12599 (1223)	12561 (1228)	12714 (1245)	12445 (1202)	12649 (1239)	12541 (1228)
Rmerge	0.01159 (0.03596)	0.01631 (0.0311)	0.01887 (0.03872)	0.02288 (0.09924)	0.01083 (0.02603)	0.01813 (0.03911)
Rmeas	0.01639 (0.05086)	0.02307 (0.04399)	0.02668 (0.05476)	0.03236 (0.1404)	0.01532 (0.03681)	0.02563 (0.05531)
<I/σ(I)>	73.58 (17.30)	83.84 (28.08)	67.7 (20.0)	41.26 (7.36)	69.31 (22.67)	74.67 (19.38)
CC(1/2)	1 (0.996)	0.999 (0.996)	0.999 (0.994)	0.999 (0.971)	0.999 (0.998)	0.999 (0.995)
ΔanomCC(1/2)	0.8 (0.2)	0.9 (0.4)	0.8	0.6 (0.1)	0.9 (0.4)	0.9 (0.3)
anomalous completeness (%)	99.9 (98.2)	94.6 (86.4)	99.9 (98.1)	100 (100)	98.4 (85.3)	98.6 (86.4)
Anomalous multiplicity	32.1 (4.7)	55.8 (2.2)	33.7 (9.0)	35.0 (10.7)	30.9 (3.7)	31.4 (2.7)
Wilson B factor (Å^2)	40.45	33.60	39.14	44.90	36.80	38.57

Table S2 Optimised anomalous scattering X-ray diffraction data statistics for iron soaked crystals.

structure	residue 141 conformation	site 1 occupancy	site 2 occupancy	site 3 occupancy	site 1-site 2 distance (Å)	ref
apo wild type	FOC	-	-	-	-	[1]
wild type 2 min. soak	FOC	0.50	0.56	-	3.90	[1]
wild type 20 min. soak	3-fold channel	0.90	0.55	0.84	3.46	[1]
apo D65A	3-fold channel	-	-	-	-	[2]
D65A 2 min. soak	3-fold channel	0.65	0.25	0.60	3.70	[2]
D65A 20 min. soak	3-fold channel	0.85	0.30	0.84	3.55	[2]
apo E141A	-	-	-	-	-	[2]
E141A 2 min. soak	-	0.50	0.20	-	3.69	[2]
E141A 20 min. soak	-	0.80	0.30	0.15	3.61	[2]
apo E141D	FOC	-	-	-	-	[2]
E141D 2 min. soak	FOC	0.90	0.40	0.45	3.49	[2]
E141D 20 min. soak	FOC	0.85	0.45	0.45	3.50	[2]

Table S3. Orientation of transfer residue 141 and occupancy of metal binding sites in *Syn*Ftn. Source: [1] Bradley, J. M.; Svistunenko, D. A.; Pullin, J.; Hill, N.; Stuart, R. K.; Palenik, B.; Wilson, M. T.; Hemmings, A. M.; Moore, G. R.; Le Brun, N. E., Reaction of O_2 with a diiron protein generates a mixed-valent Fe^{2+}/Fe^{3+} center and peroxide. . *Proc. Natl. Acad. Sci USA* **2019,** *116* (6), 2058-2067. [2] This work.

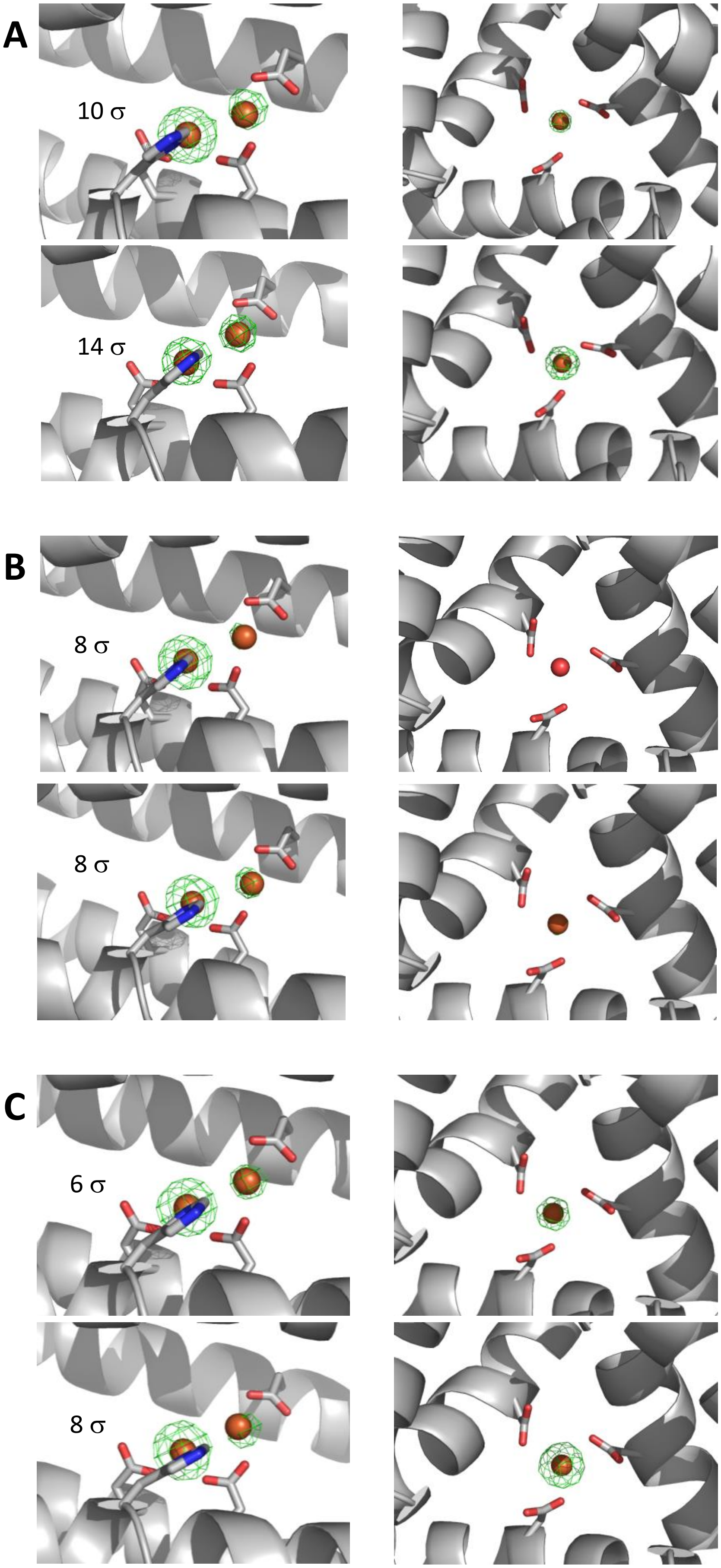
A
10 σ
14 σ
B
8 σ
8 σ
C
6 σ
8 σ

Figure S1. Anomalous difference maps for *Syn*Ftn variants E141D (A), E141A (B) and D65A (C). The upper row of each panel shows the map calculated from crystals soaked in Fe^{2+} for 2 min and the lower row for 20 min. The left hand images show iron bound at the FOC with the anomalous difference as a green mesh contoured at the indicated rmsd above the mean. Right hand images show iron in site 3 with the anomalous difference as a green mesh contoured at 6 σ above the mean.

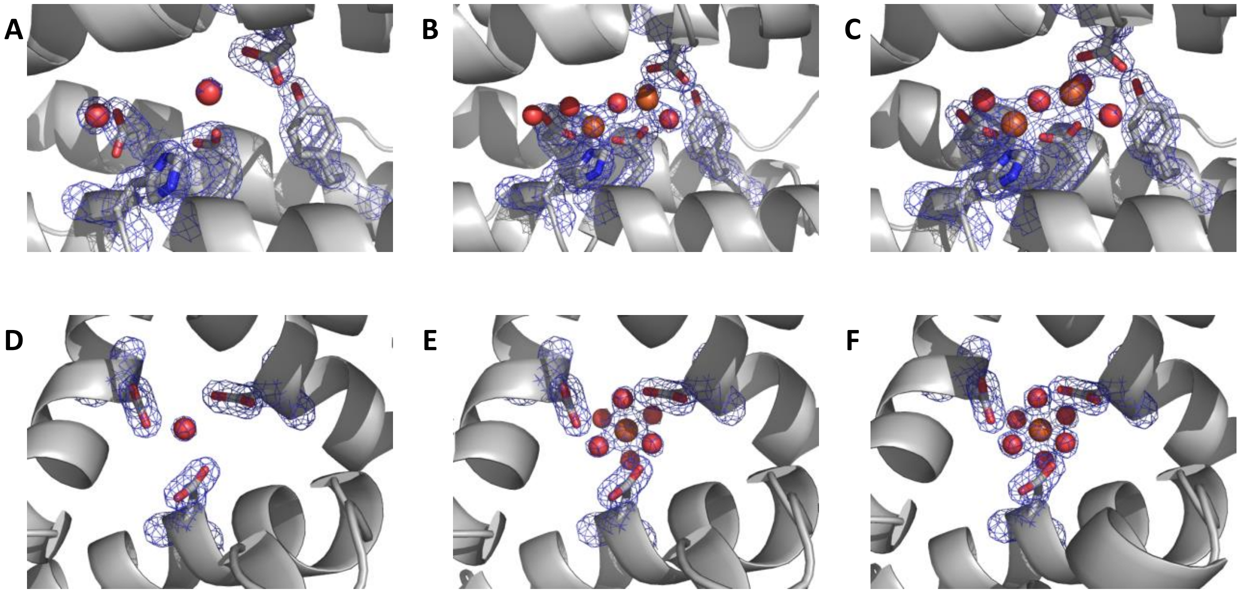

Figure S2. Iron binding to *Syn*Ftn D65A. The double difference Fourier ($2mF_o$-DF_c) map in the vicinity of the iron binding sites contoured at 1.5σ represented as a blue mesh. Metal-free (A), 2 min iron-soaked (B) and 20 min iron-soaked (C) FOC, and metal-free (D), 2 min iron-soaked (E) and 20 min iron-soaked (F) 3-fold channel are shown in cartoon representation and the side chains that constitute the catalytic centre and site 3 shown as sticks. Carbon atoms are coloured grey, oxygen in red, nitrogen in blue. Iron and water are shown as orange and red spheres, respectively. In the metal free structures, the FOC metal binding sites are vacant and that in the 3-fold channel occupied by water.

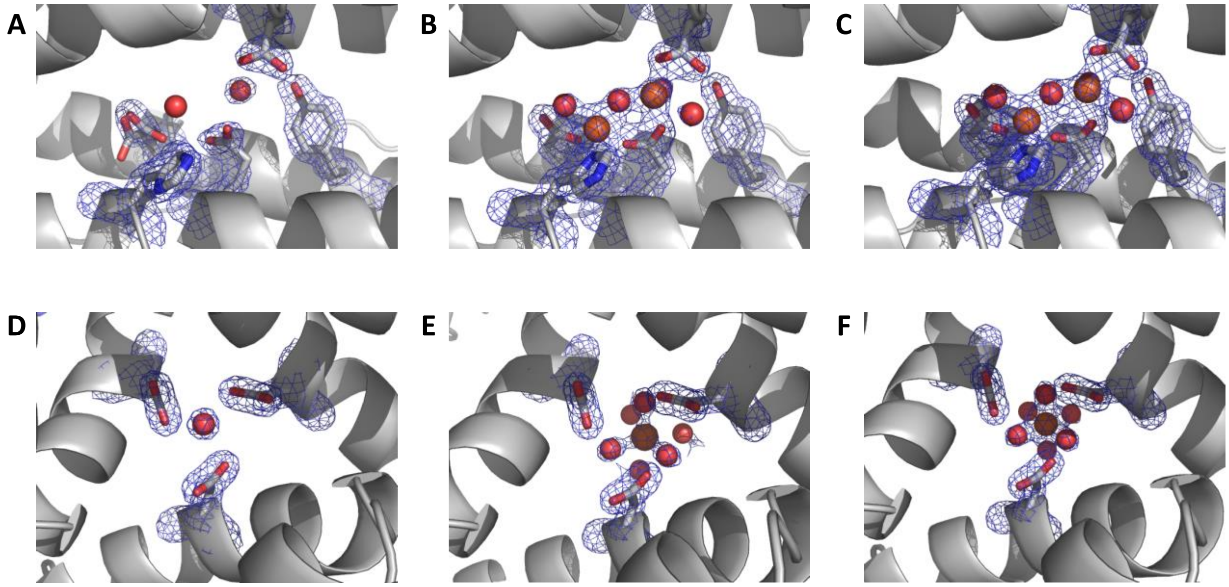

Figure S3. Iron binding to *Syn*Ftn E141D. The double difference Fourier ($2mF_o$-DF_c) map in the vicinity of the iron binding sites contoured at 1.5σ represented as a blue mesh. Metal-free (A), 2 min iron-soaked (B) and 20 min iron-soaked (C) FOC, and metal-free (D), 2 min iron-soaked (E) and 20 min iron-soaked (F) 3-fold channel are shown in cartoon representation and the side chains that constitute the catalytic centre and site 3 shown as sticks. Carbon atoms are coloured grey, oxygen in red, nitrogen in blue. Iron and water are shown as orange and red spheres, respectively. In the metal-free structures the FOC metal binding sites are vacant and that in the 3-fold channel occupied by water.

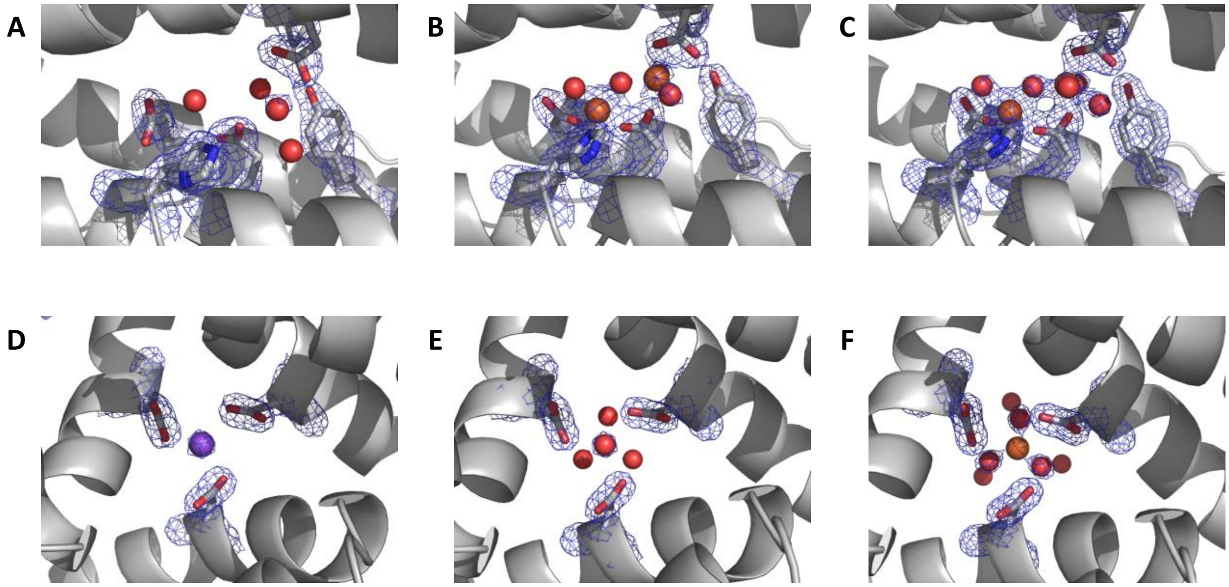

Figure S4. Iron binding to *Syn*Ftn E141A. The double difference Fourier (2mF_o-DF_c) map in the vicinity of the iron binding sites contoured at 1.5σ represented as a blue mesh. Metal-free (A), 2 min iron-soaked (B) and 20 min iron-soaked (C) FOC, and metal-free (D), 2 min iron-soaked (E) and 20 min iron-soaked (F) 3-fold channel are shown in cartoon representation and the side chains that constitute the catalytic centre and site 3 shown as sticks. Carbon atoms are coloured grey, oxygen in red, nitrogen in blue. Iron, sodium and water are shown as orange, purple and red spheres, respectively. In the metal-free structures, the FOC metal binding sites are vacant and that in the 3-fold channel occupied by sodium. In the 2 min iron soak structure the sodium ion in the 3-fold channel has been replaced by water.

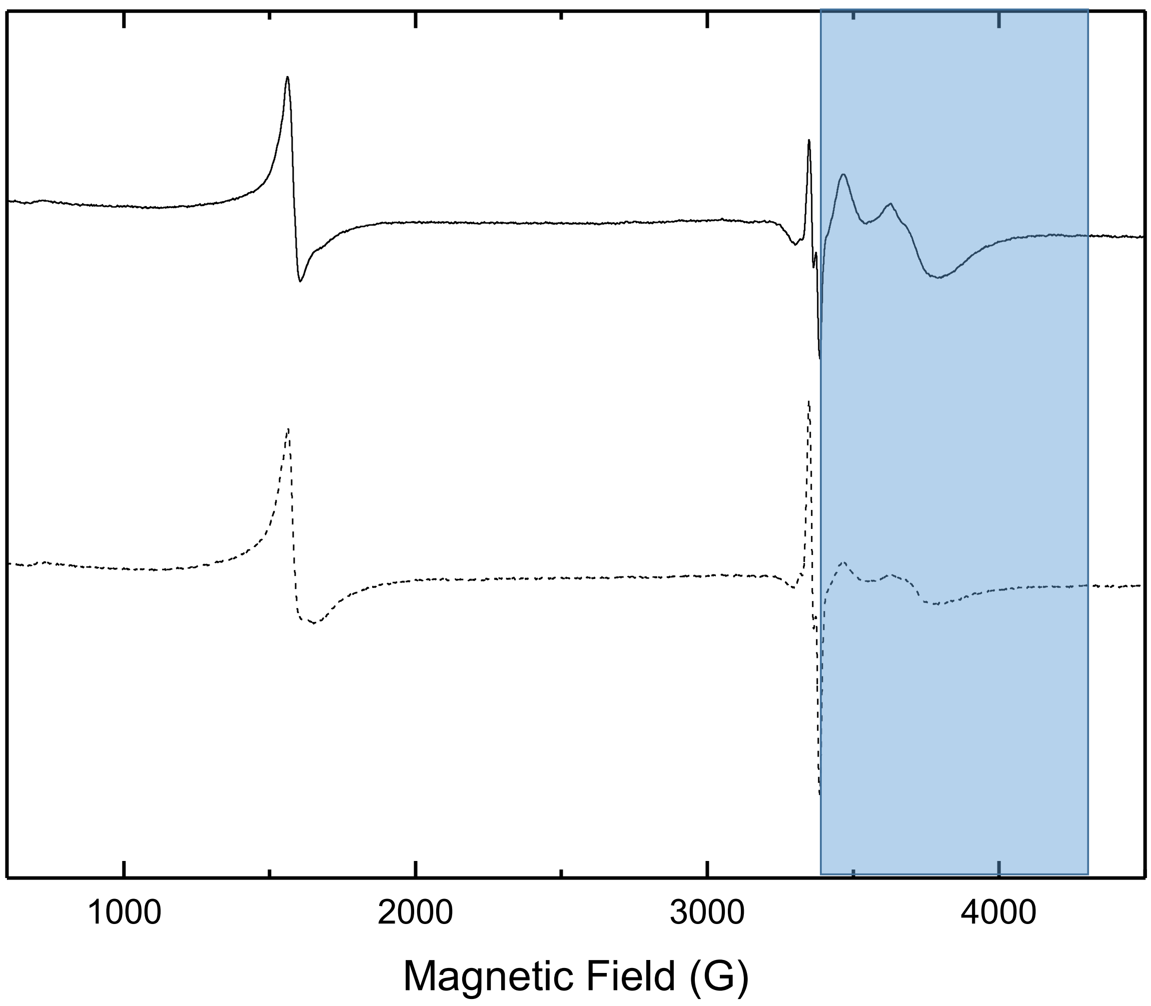

Figure S5. Electron paramagnetic resonance spectra of *Syn*Ftn. The spectrum of D65A *Syn*Ftn (upper solid trace) with that of the wild type protein (lower dashed trace) for comparison. The rhombic feature at high field, with all g values < 2, highlighted by the blue shaded area arises from the Fe^{2+}/Fe^{3+} mixed valent form of the FOC.
